# Hepatocellular carcinoma-associated hypercholesterolemia: involvement of proprotein-convertase-subtilisin-kexin type-9 (PCSK9)

**DOI:** 10.1186/s40170-018-0187-2

**Published:** 2018-10-25

**Authors:** Dipti Athavale, Surbhi Chouhan, Vimal Pandey, Shyamananda Singh Mayengbam, Snahlata Singh, Manoj Kumar Bhat

**Affiliations:** 1 Laboratory No. 6, National Centre for Cell Science, Savitribai Phule Pune University Campus, Ganeshkhind, Pune, 411 007 India; 2 Present address: Laboratory of Neuroscience, Department of Biotechnology & Bioinformatics, School of Life Sciences, University of Hyderabad, Hyderabad, Telangana 500046 India

**Keywords:** Hepatocellular carcinoma, Hypercholesterolemia, Glucose, PCSK9, Sorafenib

## Abstract

**Background:**

PCSK9 regulates low-density lipoprotein cholesterol (LDLc) level and has been implicated in hypercholesterolemia. Aberrant plasma lipid profile is often associated with various cancers. Clinically, the relationship between altered serum lipid level and hepatocellular carcinoma (HCC) has been documented; however, the underlying cause and implications of such dyslipidemia remain unclear.

**Methods:**

The present study includes the use of HepG2 tumor xenograft model to study the potential role of glucose (by providing 15% glucose via drinking water) in regulating PCSK9 expression and associated hypercholesterolemia. To support in vivo findings, in vitro approaches were used by incubating HCC cells in culture medium with different glucose concentrations or treating the cells with glucose uptake inhibitors. Impact of hypercholesterolemia on chemotherapy was demonstrated by exogenously providing LDLc followed by appropriate in vitro assays.

**Results:**

We observed that serum and hepatic PCSK9 level is decreased in mice which were provided with glucose containing water. Interestingly, serum and tumor PCSK9 level was upregulated in HepG2-tumor-bearing mice having access to water containing glucose. Additionally, elevated LDLc is detected in sera of these mice. In vitro studies indicated that PCSK9 expression was increased by high glucose availability with potential involvement of reactive oxygen species (ROS) and sterol regulatory element binding protein-1 (SREBP-1). Furthermore, it is also demonstrated that pre-treatment of cells with LDLc diminishes cytotoxicity of sorafenib in HCC cells.

**Conclusion:**

Taken together, these results suggest a regulation of PCSK9 by high glucose which could contribute, at least partly, towards understanding the cause of hypercholesterolemia in HCC and its accompanied upshots in terms of altered response of HCC cells towards cancer therapy.

**Electronic supplementary material:**

The online version of this article (10.1186/s40170-018-0187-2) contains supplementary material, which is available to authorized users.

## Background

Proprotein-convertase-subtilisin-kexin type-9 (PCSK9) is a serine protease, primarily synthesized by the liver. Following the autocatalysis in endoplasmic reticulum, it is secreted as a catalytically inert heterodimer consisting of c-terminal domain and a non-covalently bound pro-domain [1–3]. Extracellular PCSK9 binds and targets low-density lipoprotein receptor (LDLR) towards lysosomal degradation thus, restricting its recycling to the cell surface [1]. There is a tight correlation between serum PCSK9 and low-density lipoprotein cholesterol (LDLc) level in humans as PCSK9 is a post-transcriptional inhibitor of LDLR [4]. Apart from the mutations in LDLR and apolipoprotein B (ApoB), the gain of function mutation in PCSK9 gene is implicated in autosomal dominant familial hypercholesterolemia [3]. Several studies highlight the involvement of PCSK9 in imparting advantages to certain cancer types. PCSK9, also known as neural apoptosis-regulated convertase 1 (NARC1), exhibits an anti-apoptotic function in lung cancer and neuroglioma cells [5, 6]. It is demonstrated to be involved in metastasis such that its deficiency reduces melanoma metastasis due to lower circulatory cholesterol level [7]. Moreover, it induces hyperlipidemia which promotes tumor growth [8].

Hepatocellular carcinoma (HCC), the most common form of primary liver cancer, is the sixth largest cancer type based on the number of incidences worldwide [9]. It is more prevalent in Asia and accounts for 75% of all liver cancer cases [10]. Chronic viral hepatitis, history of alcohol consumption, fatty liver disorders, and metabolic syndrome led cirrhosis are the major risk factors for HCC [11]. With poor prognosis and limited treatment options, liver cancer is ranked fourth for cancer-related deaths [9, 12]. Currently, sorafenib, a multi-kinase inhibitor, is the primary chemotherapeutic agent for HCC treatment [13]. However, resistance to sorafenib is emerging and multiple mechanisms have been proposed [14].

Although hepatitis B (HBV) and C (HCV) virus infections are causative factors for HCC pathogenesis, other non-viral factors like obesity or type 2 diabetes mellitus (T2DM) are emerging as independent risk factors. Obesity or T2DM-promoted influx of nutrients (sugars, free fatty acids), pro-inflammatory cytokines, adipokines, and insulin resistance cause chronic low-grade inflammation in the liver. This can accelerate development cascade of liver diseases like non-alcoholic fatty liver disease (NAFLD), non-alcoholic steatohepatits (NASH), and cirrhosis, ultimately leading to HCC [15, 16]. Cancer cells rewire their cellular metabolism to enhance glucose uptake to support rapid reproduction [17]. Hyperglycemia, a common manifestation of diabetes or obesity, renders an advantageous access of glucose to cancer cells, thus directly or indirectly contributing to the risk and progression of tumors [18–20].

The effect of different nutrients on tissue gene expression and serum PCSK9 has been studied extensively. Various dietary lipids either did not change or lowered serum PCSK9 in healthy human subjects [21]. However, the role of glucose in the regulation of PCSK9 expression remains elusive. Considering the co-existence of HCC and metabolic syndromes which are often associated with the persistent nutritional overload, altered PCSK9 regulation is indeed a possibility. On these lines, we hypothesized that glucose could have effect on PCSK9 expression in HCC tumor which may have implications in altering LDLc level in the host. By modulating glucose availability, serum and tissue expression of PCSK9 was analyzed in the presence and absence of HCC-xenograft. In vitro studies were also focused on validating the in vivo observation and evaluating its possible consequence on the response towards chemotherapy.

## Methods

### Cell lines and cell culture

Human hepatocellular carcinoma cell lines HepG2 and Hep3B were procured from ATCC (Manassas, VA, USA) and were maintained at our in-house cell repository at National Centre for Cell Science (NCCS), Pune, India. HuH-7 cell line was obtained from Dr. Ralf Bartenschlager (University of Heidelberg, Germany). Cells were cultured in Dulbecco’s modified Eagle medium (DMEM) supplemented with 10% heat-inactivated fetal bovine serum (life technologies, CA, USA), penicillin (100 U/ml), and streptomycin (100 μg/ml) (Invitrogen Life Technologies, CA, USA) and maintained in a humidified atmosphere of 5% CO_2_ at 37 °C. Cells were intermittently checked for their typical morphology.

### Chemicals and antibodies

d-glucose, d-mannitol, glutamine, sodium pyruvate, sodium lactate, 2-deoxyglucose, crystal violet, fasentin, fatostatin, catalase, lipoprotein-deficient serum (LPDS), vinculin antibody (V9131) and thiazolyl blue tetrazolium bromide (MTT) were purchased from Sigma-Aldrich (MO, USA). Cytochalasin B was purchased from MP Biomedicals (OH, USA). Recombinant PCSK9 protein was purchased from BioLegend (CA, USA). LDLc was purchased from Lee Biosolutions (Missouri, USA) and reconstituted as per manufacturer’s instruction. PCSK9 (sc-66996), Transferrin Receptor (TfR; sc-7087), PCNA (sc-56), β-actin (sc-1615), procaspase 3 (sc-7272), SREBP-1 (sc-8984), SREBP-2 (sc-5603), GAPDH (sc-20357), Histone H1 (sc-10806), pERK (sc-7383), ERK (sc-154), Insulin (sc-8033), HRP-conjugated secondary antibodies, and sorafenib (sc-357801A) were purchased from Santa Cruz Biotechnology (CA, USA). LDLR (ab-30532) antibody was purchased from Abcam (MA, USA). Antibody for PARP (9542) was purchased from Cell Signaling Technology (MA, USA). Mouse PCSK9 antibody (AF-3985) was purchased from R&D Systems (MN, USA). Low-density lipoprotein from human plasma, DiI complex (DiI-LDL), was purchased from Life Technologies (OR, USA). BD Matrigel™ was purchased from BD Biosciences (MA, USA).

### Animal experiment

All animal experiments were performed as per the requirement and guidelines of the Committee for the Purpose of Control and Supervision of Experiments on Animals (CPCSEA), Government of India, and after obtaining permission of the Institutional Animal Ethics Committee (IAEC). Six- to 8-week-old male NOD/SCID mice were divided into four groups (*n* = 6 per group). Mice from group I and group III had access to drinking water without glucose while mice from group II and group IV were supplied with 15% glucose via drinking water throughout the experiment [19]. After 15 days, mice from group III and group IV were subcutaneously (s.c.) injected with HepG2 cells (5 × 10^6^/mouse) along with matrigel. Once the tumors were palpable, bi-dimensional measurements were taken with Vernier Caliper every third day. Tumor volume (mm^3^) was calculated with the formula (width^2^ × length) × 0.52. For glucose tolerance test (GTT), mice from group I and group II were fasted for 6 h, and blood glucose was measured with ACCU-CHEK Active glucometer (Roche diagnostics, Germany) and considered as basal glucose at 0 min. Mice were injected with glucose (3 g/ kg) intraperitoneally, and blood glucose level was measured at 30 min, 60 min, 90 min, and 120 min. At the end of the experiment, mice were fasted for 4 h and sacrificed by CO_2_ euthanasia. Subcutaneous tumor and liver were excised and stored at − 80 °C for further assays.

### Serum biochemistry

Before euthanizing the experimental mice from all the groups, blood was collected by orbital sinus puncture. Serum lipid parameters such as LDLc, total cholesterol (TC), and triglycerides (TG) were estimated as described previously [22].

### In vitro treatments

HCC cells seeded in 6-well plates were cultured in DMEM with low glucose (LG; 5 mM) or high glucose (HG; 25 mM) along with concentrations of inhibitors and substrates as indicated, and cells were processed for relevant functional assays. Plasmid expressing hPCSK9 was a kind gift from Dr. Jay Horton (UT South-western, TX, USA). HepG2 cells were transfected with empty vector or hPCSK9 plasmid using Lipofectamine 2000 (Life technologies, OR, USA) for 8 h. Post-transfection, HepG2 cells were cultured in HG for 48 h and overexpression of PCSK9 was checked by Western blotting.

### Indirect ELISA

Sera from mice or lyophilized culture media from HepG2 cells treated with LG or HG were used to coat ELISA plates at 4 °C overnight. The plates were then processed as described previously [23]. Standard curves with different concentrations of recombinant hPCSK9 and mPCSK9 were prepared to quantify concentration of secretory PCSK9.

### Quantitative reverse transcription PCR (RT-qPCR)

Total RNA from HepG2 cells and tissues was isolated with TRIzol™ (Invitrogen, CA, USA). Complementary DNA was prepared from 2 μg of total RNA as described previously [23]. Subsequently, RT-qPCR was performed using MESA GREEN SYBR MIX (Eurogentech, Belgium) with specific primer sets (Additional file 1: Table S1). The following thermal conditions were used: step 1: 95 °C for 2 min, step 2: 95 °C for 15 s, step 3: 55 °C for 20 s, and step 4: 72 °C for 20 s, and 2^−∆∆ct^ method was used to calculate fold change in gene expression.

### Immunoblotting

Whole cell or tissue samples were lysed in ice-cold RIPA buffer as described earlier [24]. Membrane, cytosolic, and nuclear fractions were prepared as described earlier [25, 26]. After protein quantitation using Coomassie Plus Protein Assay Reagent (Thermo Scientific, IL, USA), 50–100 μg of protein samples were resolved on 8–10% of SDS-PAGE and transferred to nitrocellulose membranes. Membranes were blocked and further probed with indicated primary and HRP-conjugated secondary antibodies. Immunoreactive bands were visualized using luminescence detection reagent (Santa Cruz Biotechnology, CA, USA) and quantified with ImageJ software.

### Glucose estimation

After culturing HepG2 cells for mentioned time period in DMEM with indicated glucose concentration, supernatant was collected and the residual glucose in the spent medium was estimated with GOD-POD-based glucose assay kit (Spinreact, Spain) according to the manufacturer’s instructions.

### Immunofluorescence staining

HepG2 cells were seeded on multi-well chambered slides (MP Biomedicals, OH, USA). Cells were treated as mentioned in pertinent figure legends and processed for staining as described previously [27]. Cells were analyzed under Zeiss LSM 510 microscope, and images were processed by LSM image analysis software (Carl Zeiss, Germany).

### Reactive oxygen species (ROS) measurement

Cellular ROS generation on treating cells with LG, HG, or HG + catalase was measured with 2, 7-dichlorofluorescein diacetate (DCFDA) dye. Cells were incubated with 5 μM of DCFDA at 37 °C for 30 min. The fluorescence of DCFDA was measured on FL1 channel on flow cytometer (FACS Calibur, Becton Dickinson, USA). Data were analyzed using Cell Quest Pro software (Becton Dickinson, California, USA) for 10,000 cells.

### LDL uptake assay

HepG2 cells were grown on 12-well plates or multi-well chambered slides (MP Biomedicals, OH, USA). Following the treatment of LG or HG medium or transfection of hPCSK9 plasmid, cells were processed for LDL uptake assay by either FACS or confocal microscopy as described earlier [28]. Briefly, DiI-LDL (10 μg/ml) was added at the end of the treatment for 2 h. Cells were washed with PBS and processed for either the measurement of DiI-LDL fluorescence  in FL2 channel on flow cytometer (FACS Calibur, Becton Dickinson, USA) or fixed with 3% paraformaldehyde, mounted with UltraCruz® mounting medium (Santa Cruz Biotechnology, USA), and images were acquired on Zeiss LSM 510 microscope.

### MTT assay

Cells (2 × 10^3^ cells/ well) were seeded in 96-well plates and allowed to adhere. After 24 h, cells were treated with vehicle, LDLc, or sorafenib as per experimental requirements. At the end of the treatments, medium was removed and MTT solution (1 mg/ml) was added to each well, and plates were incubated for 4 h at 37 °C. Isopropanol (100 μl) was added to solubilize formazan crystals, and absorbance was recorded at 570 nm.

### Long-term survival assay

Cells (5 × 10^2^ cells/well) were seeded in 24-well plates and treated with vehicle, LDLc, or sorafenib as mentioned in respective figure legend. After completion of treatment, medium was removed, fresh medium was added to each well and cells were cultured for another 10 days by changing medium every third day. Thereafter, cells were fixed and stained with crystal violet, and images were taken using Olympus digital camera (Tokyo, Japan).

### Statistical analysis

All data are presented as mean ± standard deviation (SD) or mean ± standard error of the mean (SEM) as mentioned. Statistical comparison was performed by unpaired two-tailed Student’s *t* test by using Sigma Plot (Systat Software Inc., CA, USA). Statistical significance was accepted at *P* < 0.05 level (*p < 0.05, **p < 0.01, ***p < 0.001).

## Results

### PCSK9 expression is increased upon supplementation of glucose in vivo

**Fig. 1:**
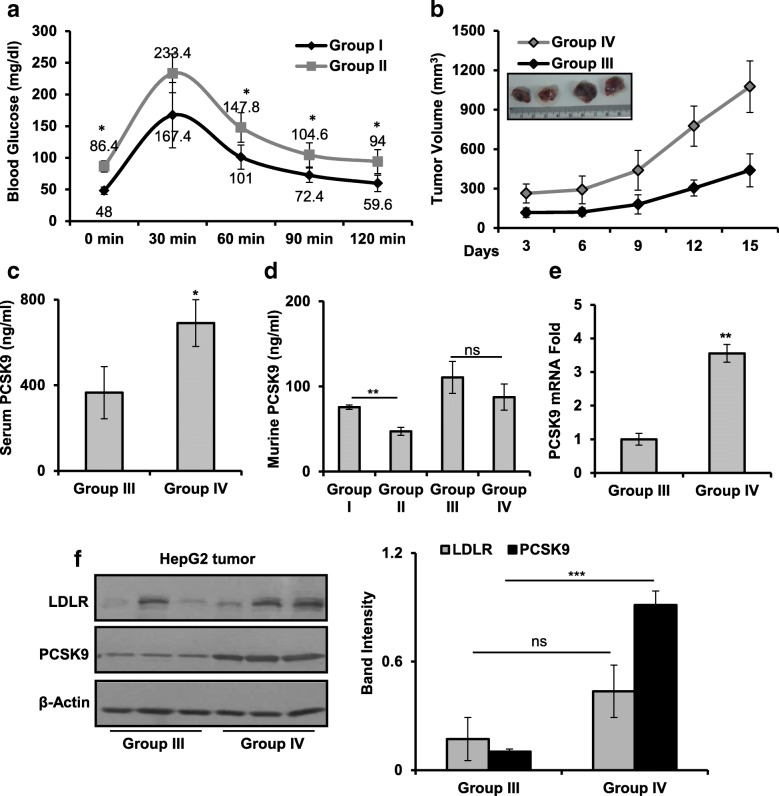
Glucose availability induces PCSK9 expression in HepG2-xenografts. **a** Intraperitoneal glucose tolerance test (GTT) in mice from group I and group II. **b** Tumor progression in group III and group IV, after injecting HepG2 cells (5 × 10^6^) subcutaneously. **c** Individual serum samples collected from group III and group IV were subjected to ELISA to quantify circulatory PCSK9 levels. **d** Individual serum samples collected from group I, group II, group III, and group IV were subjected to ELISA to quantify murine PCSK9 levels. **e** PCSK9 transcripts in three representative tumor tissue samples from group III and group IV were analyzed by qRT-PCR. **f** Tumor or liver tissue lysates from three representative samples were resolved by SDS-PAGE and LDLR, and PCSK9 protein levels were analyzed by immunoblot. Band intensities were measured by densitometry and normalized with β-actin. The results are given as means ± standard deviation; *p < 0.05, **p < 0.01, ***p < 0.001 denote significant differences in the groups; ns non-significant

To evaluate the effect of glucose on PCSK9 expression and its consequences in HCC, NOD/SCID male mice were divided into four groups. Mice from group I and group III had access to drinking water without glucose. Mice from group II and group IV were exposed to 15% glucose via drinking water throughout the experiment. Group III and group IV mice were injected with HepG2 cells subcutaneously, and tumor progression was measured. Glucose tolerance test (GTT) performed in mice from group I and group II indicated increased blood glucose levels in group II as compared to group I, implying that glucose feeding impairs blood glucose clearance and increases its availability (Fig. 1a). Additionally, glucose feeding enhanced the tumor progression in group IV, as compared to group III (Fig. 1b). Therefore, it is likely that excess glucose available to animals may be a contributory factor towards rapid proliferation of HCC cells in group IV. By ELISA, higher serum PCSK9 level was detected in mice from group IV compared to group III (Fig. 1c). Since, the antibody used in ELISA has a cross-reactivity to mouse PCSK9, to distinguish between host and graft-secreted PCSK9, murine PCSK9 level in the serum was quantified with mouse-specific antibody by ELISA. Interestingly, murine PCSK9 level was reduced in serum of non-tumor-bearing mice from group II as compared to group I (Fig. 1d). Although, increased levels of murine PCSK9 were noted in group III and group IV as compared with group I and group II, it did not change between group III and group IV (Fig. 1d). No changes in the serum insulin level were observed upon glucose feeding. Also, insulin did not induce PCSK9 expression in LG and HG medium in HepG2 cells (Additional file 2: Figure S1A and B). To seek a clear understanding of the effect of glucose on xenograft-secreted PCSK9, mRNA and protein levels of PCSK9 in tumor tissue were analyzed by qPCR and Western blot, respectively. Figure 1e shows that, mRNA expression of PCSK9 was increased significantly in tumor tissues of mice from group IV as compared to group III. Also, tumor tissue from group IV had an increase in PCSK9 protein level as compared to group III (Fig. 1f). However, in tumor tissue lysates, LDLR level remained unaltered between group III and group IV (Fig. 1f). Overall, these observations imply that elevated serum PCSK9 level detected in group IV might be contributed by HepG2-xenograft.

**Fig. 2:**
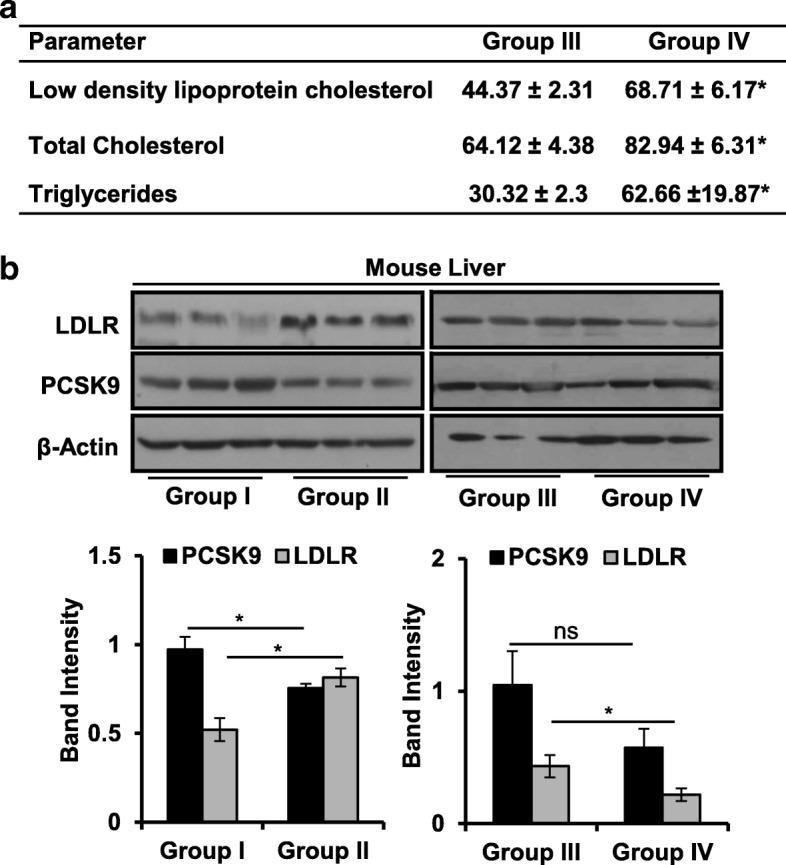
Glucose feeding differentially alters hepatic PCSK9 and LDLR protein level in presence and absence of HepG2-tumors. **a** Low-density lipoprotein cholesterol (mg/dl), total cholesterol (mg/dl), and triglycerides (mg/dl) in serum samples were estimated using kits as per the manufacturer’s instructions. **b** Mouse liver homogenates from groups I and II (left panel) and groups III and IV (right panel) were resolved by SDS-PAGE, LDLR and PCSK9 levels were analyzed by Western blot. Band intensities were measured by densitometry and normalized with β-actin. The results are given as means ± standard deviation; *p < 0.05 denote significant differences in the groups; ns non-significant

Next, lipid parameters were measured in serum collected from group III and group IV. Elevated serum LDLc, total cholesterol, and triglycerides were detected in sera of mice from group IV as compared to group III (Fig. 2a). Serum LDLc level is sensitive towards hepatic LDLR availability, and slight alteration in its level could have consequences on serum lipid profile [29, 30]. Moreover, human PCSK9 infusion in murine system effectively reduces hepatic LDLR and elevates serum LDLc [31, 32]. Therefore, LDLR protein levels in the liver and tumor tissues were analyzed. An increase in the hepatic LDLR protein was noted in glucose-fed mice from group II as compared to group I (Fig. 2b, left panel). The level of hepatic LDLR decreased in group IV as compared to group III (Fig. 2b, right panel and Additional file 2: Figure S1C and D), thus contributing for the rise in serum LDLc in group IV. However, hepatic PCSK9 protein level remained unaltered between group III and group IV (Fig. 2b, right panel). On the contrary, it decreased in group II as compared to group I (Fig. 2b, left panel) supporting our observation in Fig. 1d.

These findings imply that glucose supplementation modulates PCSK9 and LDLR levels in mice with and without HepG2 xenograft. PCSK9 derived from tumor-graft increases on glucose feeding and likely to be associated with development of hypercholesterolemia in xenograft-bearing mice from group IV as compared to group III.

### Glucose modulates PCSK9 expression in HCC cells

**Fig. 3:**
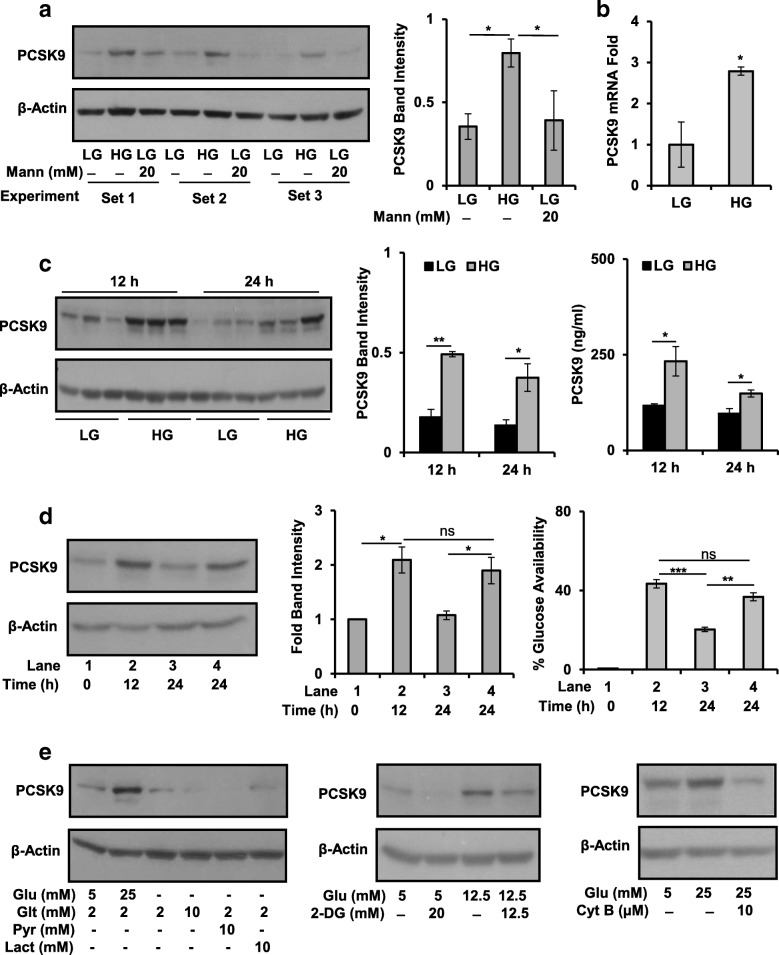
Glucose-dependent regulation of PCSK9 in HCC cells. **a** HepG2 cells were cultured in LG, HG, and LG + Mannitol (Mann) for 12 h. Expression of PCSK9 protein was examined by immunoblot in three independent sets of experiments. Band intensities were measured by densitometry and normalized with β-actin. **b** HepG2 cells were cultured in LG and HG for 12 h. PCSK9 mRNA level was quantified by qRT-PCR. **c** HepG2 cells were cultured in LG and HG for 12 h and 24 h. Immunoblot analysis was performed with total protein lysates. PCSK9 protein band intensities were quantified by densitometry and normalized to β-actin. Extracellular PCSK9 in culture supernatant was quantified by ELISA. **d** HepG2 cells were seeded in 35-mm plates and allowed to adhere. LG medium was added in all the plates for 24 h. Then, LG medium was removed and cells were incubated in HG medium for 12 h and 24 h, respectively. HG medium was replaced with fresh HG medium after 12 h, and the plate was incubated for further 12 h (lane 4). Whole cell lysates were subjected for immunoblotting to analyze expression of PCSK9. Band intensities were quantified by densitometry and normalized to β-actin. Glucose remaining in the culture supernatant was estimated at each time point. **e** HepG2 cells were treated with indicated concentrations of glucose (Glu), glutamine (Glt), pyruvate (pyr), lactate (lact) 2-deoxyglucose (2-DG), and cytochalasin B (CytB) for 12 h and expression of PCSK9 was analyzed by Western blot. Bar graphs represent mean ± SEM; *n* = 3; *p < 0.05, **p < 0.01 denote significant differences in the groups; ns non-significant

To corroborate above stated findings in vitro, human hepatoma cells, HepG2, HuH-7, and Hep3B, were cultured in increasing glucose concentrations (1 mM, 5 mM, and 25 mM) and the level of PCSK9 protein was analyzed. HepG2 and HuH-7 cells exhibited a glucose-dependent increase in PCSK9 protein level at 12 h and 24 h, respectively. Whereas in Hep3B cells, PCSK9 level decreased at higher glucose concentration at 24 h (Additional file 3: Figure S2A). Hyper osmotic stress is known to affect expression of several proteins [33]; therefore, mannitol (Mann; 20 mM) was used as an osmolarity control. PCSK9 level remained comparable to low glucose (LG; 5 mM) upon adjusting the osmolarity with mannitol suggesting that hyperosmolarity associated with high glucose (HG; 25 mM) had minimal effect on glucose-induced PCSK9 protein expression (Fig. 3a and Additional file 3: Figure S2B). Further, in HepG2 cells cultured in HG, PCSK9 mRNA level was significantly upregulated as compared to LG, at 12 h (Fig. 3b). PCSK9 being a secretory protein, its extracellular level in culture medium was measured by ELISA. A significant increase in secretory as well as intracellular PCSK9 was detected in HG as compared to LG culture condition at 12 h and 24 h (Fig. 3c). Also, the percentage of glucose availability decreased in LG and HG medium during 0 h to 24 h; however, it remained significantly higher in HG as compared to LG (Additional file 3: Figure S2C).

Having demonstrated the glucose-stimulated expression of PCSK9, its specific dependency on glucose was further evaluated. HepG2 cells when cultured in LG medium for 24 h, not only glucose availability in the medium was reduced but also cellular PCSK9 expression was decreased (Fig. 3d, lane 1 of right and left panel, respectively). Increase in the percent glucose availability and cellular PCSK9 protein expression was detected at 12 h after addition of fresh HG medium (Fig. 3d, lane 2). Similarly, replenishing fresh HG medium after 12 h and incubating HepG2 cells for the next 12 h (Fig. 3d, lane 4), resulted in a significant increase in glucose availability and cellular PCSK9 protein, as compared to 24 h of HG treatment without replenishment (Fig. 3d, lane 3). This also indicates that a decrease in the glucose availability even in HG medium reduces PCSK9 protein expression. Further, culturing HepG2 cells in LG and HG for longer time points by replenishing respective medium every 12 h indicated that PCSK9 expression was elevated in HG at all time points (Additional file 3: Figure S2D). This illustrates that restocking of fresh HG medium replenishes glucose thus causing an increase in PCSK9 protein.

To further evaluate whether PCSK9 induction is specific to glucose, HepG2 cells were cultured in various other carbon sources. Culturing HepG2 cells individually in glutamine, pyruvate and lactate did not cause any detectable alteration in PCSK9 protein expression in absence of glucose up to 12 h (Fig. 3e). A number of specific as well as non-specific glucose uptake inhibitors are reported in literature [19, 34, 35]. As shown in Fig. 3e (middle and right panel), PCSK9 level was decreased on treating HepG2 cells with 2-deoxyglucose (competitive inhibitor) or cytochalasin B (non-competitive inhibitor) for 12 h. Treatment with fasentin (GLUT1 inhibitor) resulted in the reduction in PCSK9 level in a dose-dependent manner (Additional file 3: Figure S2E). Thus, the above stated findings suggest that induction of PCSK9 expression is specific to glucose availability and uptake.

### Glucose stimulates PCSK9 expression via ROS and SREBP-1 without alteration in LDLR

**Fig. 4:**
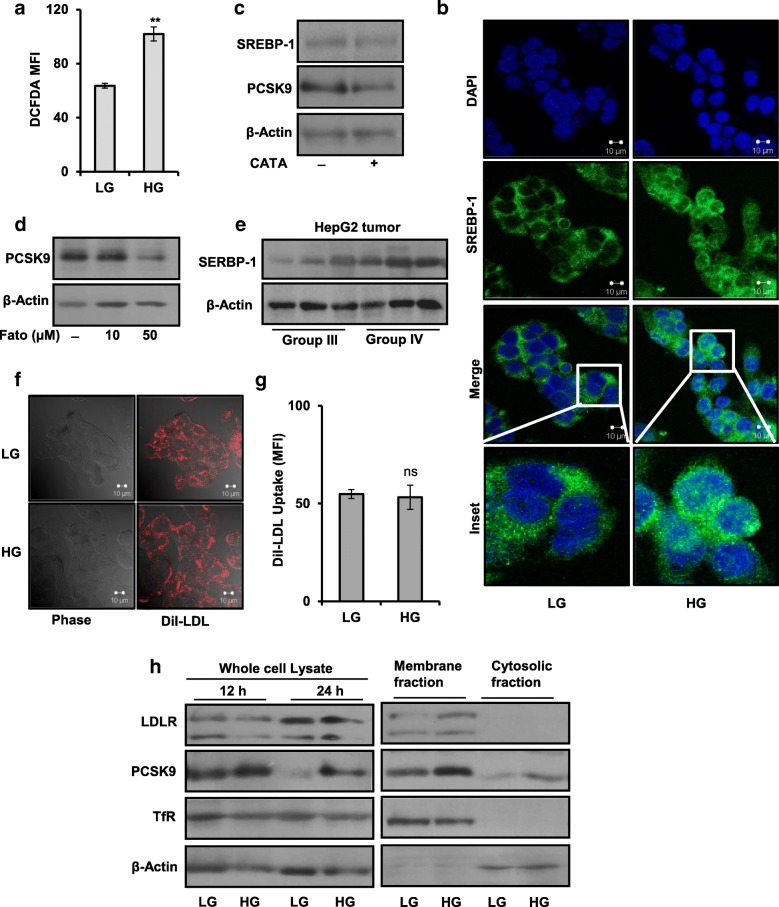
Glucose stimulates PCSK9 expression via ROS and SREBP-1 but does not alter LDLR. **a** Measurement of ROS level in HepG2 cells treated with LG and HG medium for 12 h. **b** Immunofluorecence staining of SREBP-1 in HepG2 cells cultured in LG and HG for 12 h (Scale bar = 10 μm). **c** HepG2 cells were pre-treated with 1500 U/ml of catalase (CATA) for 3 h; subsequently, HG treatment was given for 6 h. Whole cell lysates were resolved by SDS-PAGE and SREPB-1; PCSK9 and β-actin protein levels were analyzed by immunoblot. **d** HepG2 cells were pre-treated with indicated concentrations of fatostatin (Fato) for 3 h, subsequently HG treatment was given for 6 h. Whole cell lysates were resolved by SDS-PAGE. PCSK9 and β-actin protein levels were analyzed by immunoblot. **e** Tumor tissue homogenates of three representative samples from group III and group IV were resolved on SDS-PAGE, and SREBP-1 protein level was analyzed by Western blot. **f** Whole cell lysates were prepared from HepG2 cells treated with LG and HG for 12 h and 24 h, respectively, or membrane and cytosolic fractions were prepared from HepG2 cells treated with LG and HG for 24 h by replenishing respective medium after 12 h. Expression of PCSK9 and LDLR were analyzed by immunoblot. Transferring receptor (TfR) and β-actin were included in the Western blot as loading controls for membrane and cytosolic fractions. **g**, **h** HepG2 cells were incubated in LG and HG medium for 12 h and processed for DiI-LDL uptake assay either by FACS or confocal staining. All bar graphs represent means ± SEM; **p < 0.01 denotes significant differences between the groups; ns non-significant

Hyperglycemia (high glucose) is often associated with overproduction of reactive oxygen species (ROS). It is also known to induce sterol regulatory element binding protein-1 (SREBP-1) [25, 36, 37]. Transcription factors, SREBP-1 and SREBP-2, bind to the sterol response element (SRE) in the promoter region of PCSK9 gene and regulate its expression [38]. In endothelial and smooth muscle cells, inhibition of ROS, downregulates PCSK9 protein. Moreover, increased ROS level is implicated in upregulating SREBP-1 [39, 40]. Thus, involvement of ROS and SREBP-1 in glucose-induced PCSK9 expression was investigated. In HepG2 cells, HG treatment increased mean fluorescence intensity (MFI) of DCFDA dye, indicating elevated ROS level as compared to LG (Fig. 4a). Also, increased level of nuclear form of SREBP-1 was detected in HG-treated HepG2 cells as compared to LG treatment by immunofluorescence and immunoblotting (Fig. 4b and Additional file 4: Figure S3A, respectively). However, no noticeable change was detected in SREBP-2 protein level in nuclear fraction of HG-treated HepG2 cells as compared to LG treatment (Additional file 4: Figure S3B). Further, if ROS regulates PCSK9 via SREBP-1, curtailing ROS level would downregulate SREBP-1 and PCSK9. Pre-treatment of HepG2 cells with catalase (CATA), a ROS scavenger [41], decreased MFI of DCFDA dye in HG (Additional file 5: Figure S4A and B). Subsequently, it also decreased SREBP-1 and PCSK9 protein level (Fig. 4c). To analyze the involvement of SREBP-1 in PCSK9 regulation, HepG2 cells were treated with fatostatin, a pharmacological inhibitor of SREBP-1 activation [37]. PCSK9 protein level was decreased in HepG2 cells treated with fatostatin in dose-dependent manner (Fig. 4d). Further, in agreement with the in vitro data, SREBP-1 level was upregulated in tumor tissue lysates of mice from group IV in comparison with group III (Fig. 4e).

Subsequently, to study if glucose regulates LDLR via PCSK9, LDLR level and its activity under LG and HG culture conditions were checked by immunoblotting and DiI-LDL uptake assay, respectively. No significant changes in LDLR level or DiI-LDL uptake were detected in HepG2 cells cultured under LG and HG for 12 h (Additional file 6: Figure S5A and Fig. 4f, g, respectively). Further, expression of LDLR protein, in whole cell lysates and membrane-cytosolic fractions, were analyzed at different time points by Western blot. Despite changes in the level of PCSK9 in whole cell lysate or membrane-cytosolic fractions, changes in LDLR level were not detected at 12 h and 24 h (Fig. 4h). However, overexpressing human PCSK9 in HepG2 cells by transient transfection caused reduction in LDLR level and DiI-LDL uptake as compared to control (Additional file 6: Figure S5B and C) [1, 32]. This observation suggested that extracellular PCSK9 present in HG medium is insufficient to induce LDLR degradation and, when overexpressed, it reduces LDLR level. Collectively, these results indicate a potential role of glucose stimulated ROS and SREBP-1 in PCSK9 regulation.

### Extracellular LDLc decreases efficacy of sorafenib

**Fig. 5:**
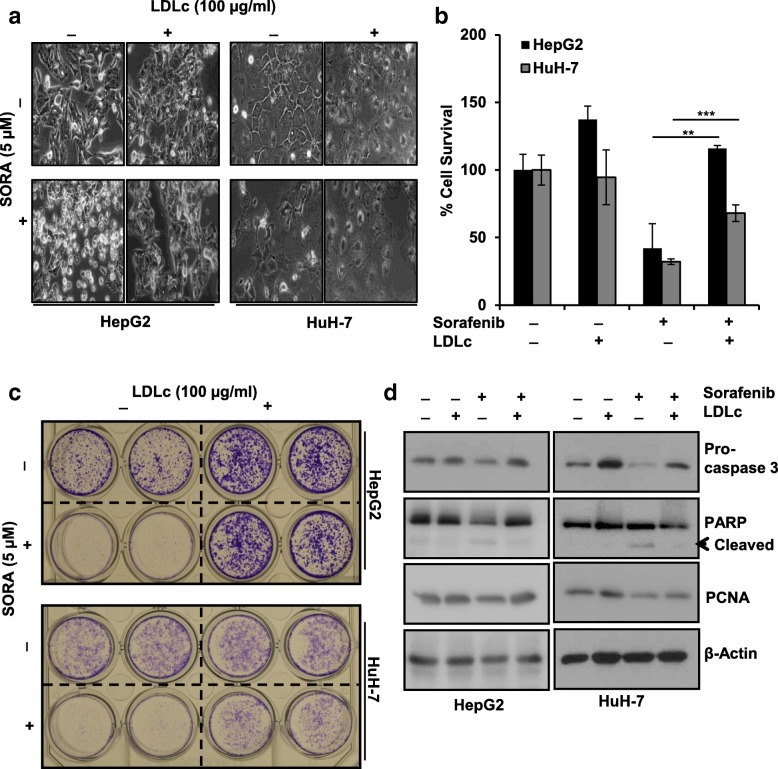
Extracellular LDLc decreases efficacy of sorafenib. HepG2 and HuH-7 cells were seeded. After adherence, they were pretreated with LDLc (100 μg/ml) in 5% LPDS for 24 h, followed by 5 μM of sorafenib (SORA) treatment for the next 48 h in the presence or absence of LDLc as indicated and processed as follows: **a** Cell images were taken by Olympus (DP-71) camera (magnification × 20). **b** Percentage of cell survival was determined using MTT assay. Vehicle-treated cells served as control and OD values of control were considered as 100% viable. Error bars represent variations within the wells of an experiment. **c** Long-term cell survival assay. **d** Whole cell lysates were resolved on SDS-PAGE, subjected to immunoblot analysis and expression of procaspase-3, cleaved PARP; PCNA were analyzed. β-Actin was used as loading control. Bar graph represents means ± standard deviation; **p < 0.01, ***p < 0.001 denotes significant differences between the groups

Rapidly proliferating cancer cells satisfy their lipid and cholesterol requirements by either increasing the uptake of dietary lipids or reprogramming intracellular lipid biogenesis. Lipid droplets (LD) which consist of excessive lipids and cholesterol are stored in cancer cells and contribute to chemo-resistance [42]. Very few studies have investigated impact of elevated extracellular lipid parameters on chemotherapy. In the present in vivo study, development of hypercholesterolemic phenotype impelled us to investigate the effect of elevated lipid parameters on the efficacy of sorafenib in HCC cells. To mimic hypercholesterolemic condition in vitro, cells were provided with LDL cholesterol exogenously and evaluated for the response to sorafenib [43]. Based on initial screening experiments and literature, sorafenib (5 μM) and LDLc (100 μg/ml) concentrations were selected for HepG2 and HuH-7 cells (Additional file 7: Figure S6). Upon sorafenib treatment, survival increased significantly in LDLc exposed cells compared to cells without LDLc exposure (Fig. 5a, b). In long-term survival assay, pre-treatment and presence of LDLc during sorafenib treatment resulted in the survival of more number of cells as compared to sorafenib treatment alone, thus complementing the protective function of LDLc on sorafenib-induced cell death (Fig. 5c). Further, to check molecular events, cells, with or without pre-exposure to LDLc, were treated with sorafenib in the presence and absence of LDLc for 48 h and hallmarks of apoptosis were analyzed. Downregulation of procaspase 3 level and increased PARP cleavage were detected in the lysates of cells treated with sorafenib as compared to LDLc exposed cells treated with sorafenib. PCNA level in sorafenib-treated cells was decreased as compared to LDLc exposed cells treated with sorafenib (Fig. 5d). Inhibition of RAF/MEK/ERK pathway has been linked to the anti-proliferative action of sorafenib [11]. Additionally, LDLc has been shown to activate ERK by promoting phosphorylation of its upstream activators like Raf and MEK [43–46]. Thus, to understand whether LDLc decreases toxicity of sorafenib by interfering with signaling pathways, activation of ERK was checked. Increased level of pERK was detected in LDLc-exposed sorafenib-treated cells as compared to sorafenib treatment alone, implying that extracellular LDLc can abrogate sorafenib-dependent signal transduction (Additional file 8: Figure S7A and B). These observations indicate that hypercholesterolemia can hamper sorafenib-induced cell killing and promote HCC cell survival.

## Discussion

In the recent past, PCSK9 has drawn attention because of its role in dyslipidemia and proposition of it being a target in the management of hypercholesterolemia associated with cardiovascular disease [47]. Nutritional status, pertaining to either fasting and feeding or various dietary interventions, has been reported to alter circulatory PCSK9 level in humans [48, 49]. Also, a study by Cariou et al. indicated an obvious relation between sugar-rich diet and serum PCSK9 level [46]. In the present study, to mimic the elevated blood glucose condition; drinking water containing glucose was provided, and its impact on PCSK9 expression was studied in HepG2-xenografted mice. Analyzing serum PCSK9 in tumor-bearing mice revealed that glucose feeding significantly upregulated its level. Since the mRNA and protein levels of PCSK9 were elevated in tumor tissues of glucose provided, tumor-bearing mice than the tumor-bearing mice without glucose feeding, the transcriptional regulation of PCSK9 by glucose is likely to be responsible for the enhanced serum level of PCSK9. Hyperinsulinemia is often linked with sugar-rich diet feeding [50, 51]; however, in present study, serum insulin levels did not alter upon glucose feeding which concurs with earlier reports [52, 53]. It should also be noted that even though insulin induced PCSK9 expression in rat/mouse hepatoma cells and primary hepatocytes [51, 54], it exhibited either neutral or inhibitory effect on serum PCSK9 in human subjects [55, 56]. Therefore, together with our observations, in this report, it is unlikely that insulin contributes to any alteration in PCSK9 expression in HepG2 xenograft.

Tumor-originated secretions of hormones, peptides, and cytokines can give rise to disorders referred as paraneoplastic syndromes (PNS) which can significantly affect clinical outcomes [57]. Various studies report abnormal lipid level in cancer patients and hyper/hypocholesterolemia can be considered as a consequence of certain cancer types [58]. Although detail studies are lacking, few clinical reports indicate reduced levels of total cholesterol in HCC patients with hepatitis virus infection [59, 60], while hypercholesterolemia was documented as PNS in HCC subjects leading to poor survival rates [61, 62]. In agreement with the latter, our in vivo data reveals that the presence of HepG2-tumor induces hypercholesterolemia upon glucose supplementation by reducing hepatic LDLR level in graft-bearing mice. However, an increase in serum PCSK9 concentration did not alter LDLR level in HepG2-tumor, highlighting the specificity of PCSK9 towards hepatic LDLR [63].

In murine physiology, nutritional status of an animal has been shown to regulate hepatic PCSK9 [51]. Therefore, in the work outlined here, it is essential to analyze whether glucose feeding modulates endogenous (murine) PCSK9 in both presence and absence of tumor. We report that glucose supplementation significantly reduces serum and hepatic PCSK9 protein level in NOD/SCID mice, devoid of HepG2-xenograft (group II vs. group I). Our observation correlates fairly well with the report by Dong et al., in which feeding fructose-rich diet to metabolically normal C57BL/6J mice reduced hepatic PCSK9 at transcript and protein level [53]. Put together, these findings suggest that changes in PCSK9 protein, in response to the carbohydrate-rich diet, is independent of strain of mouse used as, NOD/SCID and C57BL/6J mice are metabolically different [64]. In contrast to the minimum changes in hepatic LDLR as reported by Dong et al., in the present study, upregulation in hepatic LDLR protein was noted in mice without tumor-graft on glucose feeding. This difference can be due to either extended duration (2 months) of glucose feeding or differential response of liver towards glucose feeding as compared to 3 weeks of fructose feeding. Further, consistent with earlier study [8], in this work, we report increase in serum levels of murine PCSK9 in presence of tumor as compared to mice without tumor (Fig. 1d). Together with the previous literature and our observations regarding effect of sugar on PCSK9 expression, it can be concluded that, in mice physiology, high-sugar/carbohydrate feeding decreases PCSK9 protein. This strengthens our hypothesis that rather than the endogenous PCSK9, the PCSK9 secreted by HepG2-grafts is responsible for increased serum PCSK9 in mice, when glucose was provided and may be involved in reducing hepatic LDLR level in the host. The findings reported here do not exclude the involvement of other post-translational regulators of LDLR like IDOL (inducible degrader of LDL receptor). Liver X receptor (LXR) regulates expression of IDOL and glucose being an inducer of LXR; the role of glucose-LXR-IDOL axis cannot be ruled out [65, 66]. Thus, further studies are required to reveal such association if any.

In vitro studies in the present work also concur with the in vivo observation that PCSK9 expression is induced by glucose availability. Considering the exacerbated glucose uptake property of cancer cells, high glucose availability was maintained by culturing HCC cells in HG medium and expression of PCSK9 was compared with cells cultured in LG medium. In HepG2 and HuH-7 cells, treatment of HG medium, increased PCSK9 protein expression while, in Hep3B cells, downregulation of PCSK9 protein, in glucose abundance condition was noted. Decrease in PCSK9 level under HG-condition may be a consequence of viral genome integration in Hep3B cells [67] and needs further confirmation. This proposition gained strength from a study which reported a significantly decreased PCSK9 expression in HCC tissue samples, and majority of them were acquired from hepatitis virus B/C-infected HCC patients [68].

Hyperglycemia induced ROS generation, can regulate several signaling pathways, and has also been linked with complications in diabetes and obesity [69]. As, in the present study, both  ROS generation and nuclear translocation of SREBP-1 were enhanced in HG-treated HepG2 cells, involvement of glucose-ROS-SREBP-1 link may be a possibility in PCSK9 regulation. Hydrogen peroxide formed by the action of cellular superoxide dismutase is further converted to water and oxygen by catalase enzyme [41]. The process of proteolysis to form a mature form of SREBP-1 from precursor is blocked by fatostatin [70]. By employing inhibitor of ROS generation (catalase) and SREBP-1 activation (fatostatin), the present study points towards a role of ROS and SREBP-1 in PCSK9 regulation under HG which warrants further evaluation.

Metabolic rewiring supports not only proliferation of tumor cells but also the enzymes involved in these networks confer therapeutic resistance to cancer cells [71]. In addition to these peculiar cellular modifications, host-derived factors which are induced by tumor can also amend chemotherapeutic response. For instance, our recent studies showed that resistin, which is primarily secreted by tumor-infiltrated host macrophages, arrests colon cancer cells in G1 phase and hampers 5-fluorouracil uptake [23]. Similarly, in the present study, tumor-induced hypercholesterolemia in the host can have an implication in chemotherapy. Mounting evidences support the idea that cholesterol-rich extracellular milieu imparts proliferative and aggressive phenotype to cancer cells [22, 45, 46]. Moreover, we have earlier reported that cholesterol can affect the functional outcome of anticancer drugs in various cancer cell types [72, 73]. Thus, considering the profound effect of cholesterol in cancer cell growth and response towards chemotherapy, it is clinically relevant to investigate the effect of extracellular LDLc on efficacy of sorafenib. Studies have tried to find a mechanistic association between lipid-enriched cellular pathways and low response rate to cancer therapy. Plasma LDLc is uptaken by LDLR through receptor-mediated endocytosis, and indeed silencing LDLR potentiates conventional chemotherapy [74]. A shift towards de novo lipogenesis was reported to be involved in tumor relapse after sorafenib withdrawal [75]. Adding to these findings, we report elevated extracellular LDLc impairs the efficacy of sorafenib towards HCC cells. Sorafenib as well as its metabolites have strong binding affinities to plasma proteins such as human serum albumin, α and β-gluobulins, and LDL. Binding of sorafenib to plasma proteins is implicated in limiting its uptake and pharmacological/ toxicological responses [76, 77]. Serum PCSK9 level is positively correlated with plasma LDL [4]. In the present report, increase in LDL fraction due to altered PCSK9 level or hypercholesterolemia (independent of PCSK9 signaling) would increase the probability of sorafenib binding to LDL, thus reducing the fraction of drug available for uptake by the tumor cells.

Collectively, these observations suggest that intracellular lipogenic networks as well as lipid-rich extracellular milieu can influence chemotherapeutic outcome in cancer cells. Further detailed mechanistic studies regarding LDLc promoted diminished sensitivity of HCC cells to sorafenib would help in strategizing newer combination therapies in the treatment of HCC.

## Conclusions

**Fig. 6:**
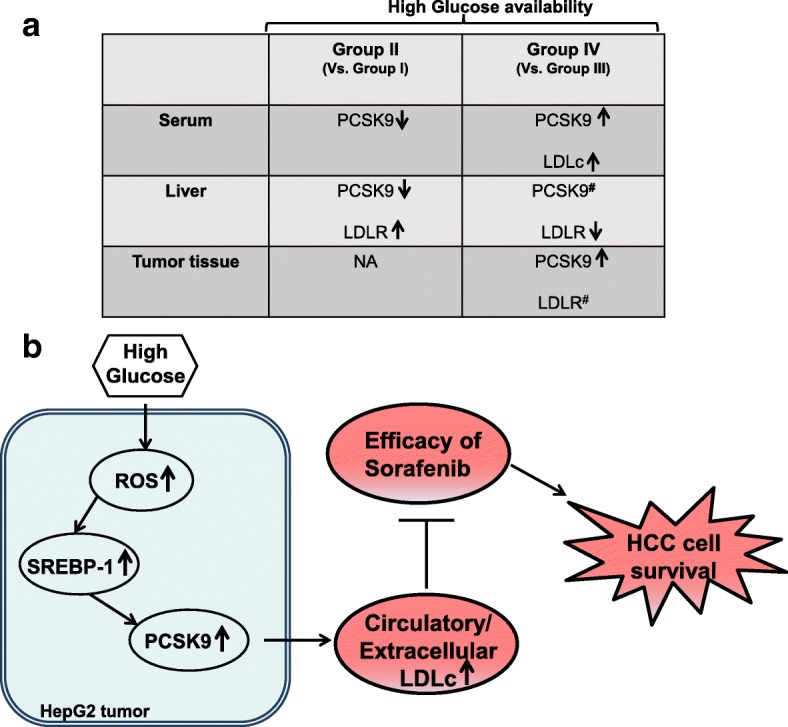
Schematic illustration. **a** Table summarizes levels of LDLc and PCSK9 in serum, LDLR and PCSK9 protein in liver and tumor tissues of mice from group II and group IV when compared with group I and group III respectively. NA denotes not applicable, and the number sign denotes non-significant change. **b** The proposed model demonstrate HG-driven changes in HepG2 tumor leading to increased expression of PCSK9 and associated hypercholesrolemia which can further lead to HCC cell survival by decreasing the efficacy of sorafenib

Overall, this study illustrates tumor-secreted PCSK9 is induced by glucose availability, and its possible involvement in causing hypercholesterolemia in HCC. As shown in the schematic table (Fig. 6a), upon glucose feeding, PCSK9 level in serum as well as in tumor tissue was elevated in mice with tumor-graft (group IV) as compared to tumor-bearing mice without glucose feeding (group III). Also, group IV had a higher serum LDLc, partially owing to the decrease in hepatic LDLR level in these mice as compared to group III. Mice from group II had decreased levels of serum and hepatic PCSK9 while upregulated level of hepatic LDLR protein when compared with mice from group I. Overall, these observations would help in understanding the association between abnormal plasma lipid level and HCC in humans. Additionally, a specific interrelation among the etiology of HCC, blood glucose, and PCSK9 needs to be explored in future. Figure 6b demonstrates a proposed model, linking glucose-induced expression of PCSK9 to its upstream regulators; ROS and SREBP-1 and downstream consequence like hypercholesterolemia can decrease efficacy of sorafenib in HCC cells. Therefore, controlling systemic cholesterol level, prior to sorafenib treatment, may have clinical implications in HCC patients.

## Additional files

Additional file 1: **Table S1.** Primer pair sets (DOCX 13 kb)

Additional file 2: **Figure S1.** Effect of glucose feeding on serum insulin and hepatic LDLR levels. (DOCX 120 kb)

Additional file 3: **Figure S2.** Glucose specificity of PCSK9 regulation (DOCX 114 kb)

Additional file 4: **Figure S3.** Effect of glucose on SREBP-1 and SREBP-2 (DOCX 50 kb)

Additional file 5: **Figure S4.** Effect of catalase on ROS generation in HG (DOCX 21 kb)

Additional file 6: **Figure S5.** Effect of glucose and PCSK9 overexpression on LDLR (DOCX 118 kb)

Additional file 7: **Figure S6.** Screening of sorafenib and LDLc concentration (DOCX 76 kb)

Additional file 8: **Figure S7.** Effect of LDLc on sorafenib mediated signaling (DOCX 112 kb)

## Abbreviations

2-DG: 2-Deoxyglucose
CATA: Catalase
CytB: Cytochalasin B
Fato: Fatostatin
Glt: Glutamine
Glu: Glucose
HCC: Hepatocellular carcinoma
HG: High glucose
Lact: Lactate
LDLc: Low-density lipoprotein cholesterol
LDLR: Low-density lipoprotein receptor
LG: Low glucose
Mann: Mannitol
PCSK9: Proprotein-convertase-subtilisin-kexin type-9
Pyr: Pyruvate
ROS: Reactive oxygen species
SORA: Sorafenib
SREBP-1: Sterol regulatory element binding protein
