# Supplementary material for: Hepatocellular carcinoma-associated hypercholesterolemia: involvement of proprotein-convertase-subtilisin-kexin type-9 (PCSK9)
Source: Cancer Metab. 2018 Oct 25;6:16. doi: 10.1186/s40170-018-0187-2 (PMC6201570; doi:10.1186/s40170-018-0187-2)
Supplement: Supplementary file 1 — Table S1. Primer pair sets (DOCX 13 kb) [file 40170_2018_187_MOESM1_ESM.docx]

**Additional File 1: Table S8 Primer pair sets**

| **Oligo Name** | **Forward** | **Reverse** |
| --- | --- | --- |
| **hPCSK9** | GAACCTGGAGCGGATTACCC | TTGCTGGCCTGTCTGTGGAA |
| **mPCSK9** | GCTTCTGCTCCAGAGGTCATC | TGTGAGGTCCCACTCTGTGA |
| **hLDLR** | GCTCTGTCCATTGTCCTCCC | CATCTGTCTCGAGGGGTAGC |
| **mLDLR** | ACAGCCTAGAGAAGTCGACAC | CTGGACTTGGTGGGACACTG |
| **18S rRNA** | GGCCCTGTAATTGGAATGAGTC | CCAAGATCCAACTACGAGCTT |
| **β-Actin** | CCAACCGCGAGAAGATGACC | GGAGTCCATCACGATGCCAG |
