# Supplementary material for: Hepatocellular carcinoma-associated hypercholesterolemia: involvement of proprotein-convertase-subtilisin-kexin type-9 (PCSK9)
Source: Cancer Metab. 2018 Oct 25;6:16. doi: 10.1186/s40170-018-0187-2 (PMC6201570; doi:10.1186/s40170-018-0187-2)
Supplement: Supplementary file 2 — Figure S1. Effect of glucose feeding on serum insulin and hepatic LDLR levels. (DOCX 120 kb) [file 40170_2018_187_MOESM2_ESM.docx]

**7Hepatocellular carcinoma associated hypercholesterolemia: Involvement of proprotein-convertase-subtilisin-kexin type-9 (PCSK9)**

**Authors:** Dipti Athavale^1^, Surbhi Chouhan^1^, Vimal Pandey ^1, #^ Shyamananda Singh Mayengbam^1^, Snahlata Singh^1^, Manoj Kumar Bhat^1,*^

**Authors’ affiliations:**

^1^National Centre for Cell Science, Savitribai Phule Pune University Campus, Ganeshkhind, Pune 411 007, India

^#^Laboratory of Neuroscience, Department of Biotechnology & Bioinformatics, School of Life Sciences, University of Hyderabad, Hyderabad- 500046, Telangana, India

**Additional File 2: Figure S1**


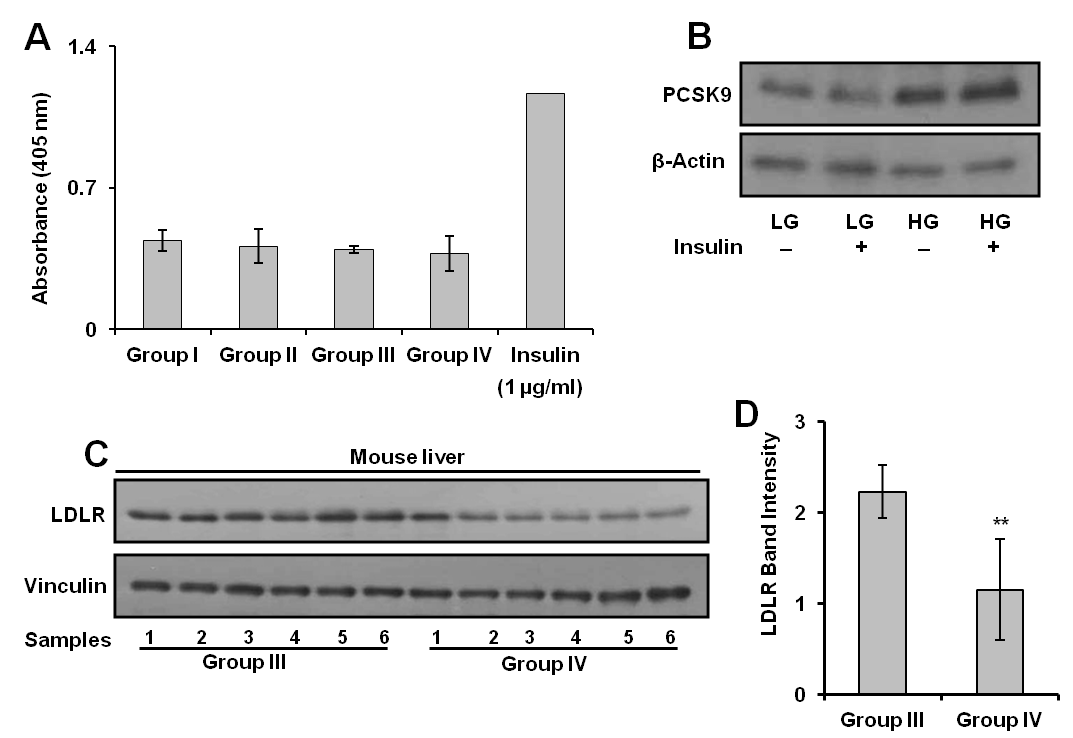


**Figure S1: Insulin induced changes in PCSK9. a** Individual serum samples collected from Group I, Group II, Group III and Group IV were subjected to ELISA to quantify Insulin levels. The results are given as means ± standard deviation. **b** HepG2 cells were cultured in LG and HG in presence or absence of insulin (100 nM) for 12 h. Expression of PCSK9 was analysed by Western blot. **c, d** Liver tissue lysates from Group III and Group IV (n=6) were resolved by SDS-PAGE and LDLR protein levels were analyzed by Western Blot. LDLR band intensities were measured by densitometry and normalized with vinculin. The results are given as means ± standard deviation; **p < 0.01, denote significant differences between the groups.
