# Supplementary material for: Hepatocellular carcinoma-associated hypercholesterolemia: involvement of proprotein-convertase-subtilisin-kexin type-9 (PCSK9)
Source: Cancer Metab. 2018 Oct 25;6:16. doi: 10.1186/s40170-018-0187-2 (PMC6201570; doi:10.1186/s40170-018-0187-2)
Supplement: Supplementary file 3 — Figure S2. Glucose specificity of PCSK9 regulation (DOCX 114 kb) [file 40170_2018_187_MOESM3_ESM.docx]

**Additional File 3: Figure S2**

**
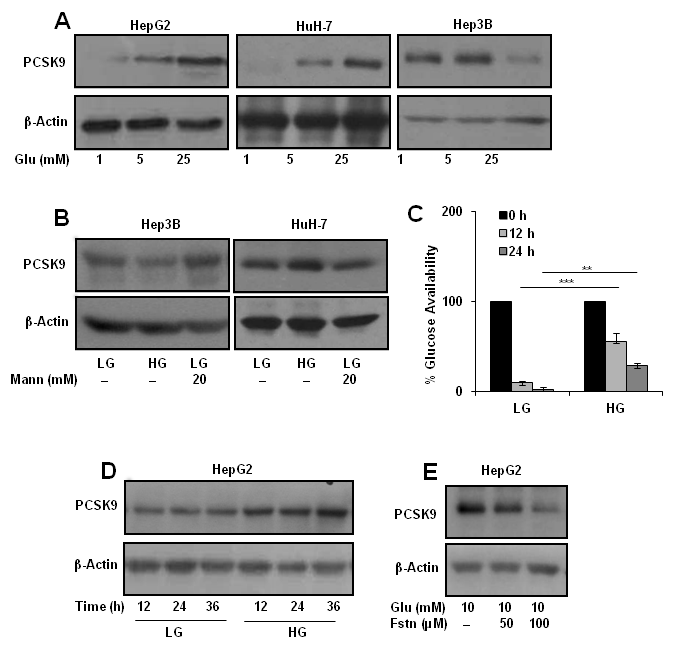
**

**Figure S2: Glucose specificity of PCSK9 regulation. a** HepG2 (12 h) and Hep3B, HuH-7 cells (24 h) were cultured in indicated concentrations of glucose (Glu) and expression of PCSK9 protein was analyzed by Western blot. **b** Hep3B and HuH-7 cells were cultured in LG, HG and LG + mannitol (Mann) for 24 h and expression of PCSK9 was examined by immunobltting. **c** HepG2 cells were cultured in LG and HG for 12 h and 24 h. Glucose remaining in the spent medium was estimated by kit. **d** HepG2 cells were cultured in LG and HG medium for indicated time period. Respective media were replenished after every 12 h. Expression of PCSK9 was analysed by immunoblot. **e** HepG2 cells were treated with indicated concentrations of Glu and fasentin (ftsn) and expression of PCSK9 was analyzed by Western blot. Bar graph represents mean ± SEM; n=3; **p<0.01, ***p<0.001 denote significant differences in the groups.
