# Supplementary material for: Hepatocellular carcinoma-associated hypercholesterolemia: involvement of proprotein-convertase-subtilisin-kexin type-9 (PCSK9)
Source: Cancer Metab. 2018 Oct 25;6:16. doi: 10.1186/s40170-018-0187-2 (PMC6201570; doi:10.1186/s40170-018-0187-2)
Supplement: Supplementary file 4 — Figure S3. Effect of glucose on SREBP-1 and SREBP-2 (DOCX 50 kb) [file 40170_2018_187_MOESM4_ESM.docx]

**Additional File 4: Figure S3**

**
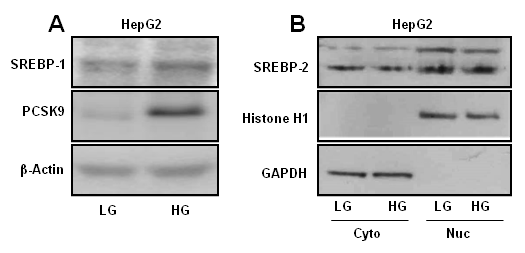
**

**Figure S3: Effect of glucose on SREBP-1 and SREBP-2.** HepG2 cells were seeded and treated with LG and HG for 12 h and processed as follows **a** Whole cell lysates were resolved on SDS-PAGE and expression of SREBP-1 and PCSK9 were analyzed by Western blot. **b** Cytosolic and nuclear fractions were prepared and expression of SREBP-2 was analysed. Histone H1 and GAPDH were included in the Western blot as loading controls for nuclear and cytosolic fractions respectively.
