# Supplementary material for: Hepatocellular carcinoma-associated hypercholesterolemia: involvement of proprotein-convertase-subtilisin-kexin type-9 (PCSK9)
Source: Cancer Metab. 2018 Oct 25;6:16. doi: 10.1186/s40170-018-0187-2 (PMC6201570; doi:10.1186/s40170-018-0187-2)
Supplement: Supplementary file 5 — Figure S4. Effect of catalase on ROS generation in HG (DOCX 21 kb) [file 40170_2018_187_MOESM5_ESM.docx]

**Additional File 5: Figure S4**


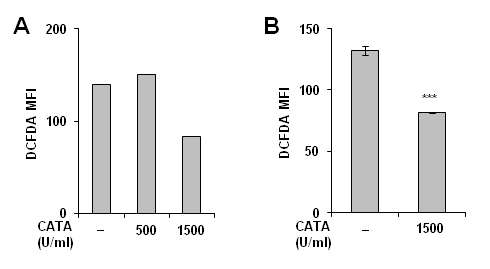


**Figure S4: Effect of catalase on ROS generation in HG.** **a and b** HepG2 cells were seeded in 12-well plates. Next day, they were pre-treated with indicated concentrations of catalase (CATA) for 3 h followed by incubation in HG medium for 3 h and cells were processed for ROS measurement by FACS. Bar graph in *panel B* represents mean intensity ± standard deviation; ***p<0.001
