# Supplementary material for: Hepatocellular carcinoma-associated hypercholesterolemia: involvement of proprotein-convertase-subtilisin-kexin type-9 (PCSK9)
Source: Cancer Metab. 2018 Oct 25;6:16. doi: 10.1186/s40170-018-0187-2 (PMC6201570; doi:10.1186/s40170-018-0187-2)
Supplement: Supplementary file 6 — Figure S5. Effect of glucose and PCSK9 overexpression on LDLR (DOCX 118 kb) [file 40170_2018_187_MOESM6_ESM.docx]

**Additional File 6: Figure S5**

**
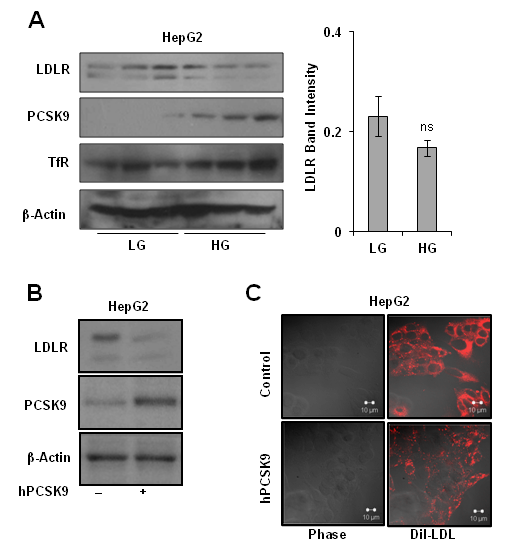
**

**Figure S5: Effect of glucose and PCSK9 overexpression on LDLR. a** Total protein was isolated from HepG2 cells treated with LG and HG medium for 12 h and LDLR protein level was examined by immunoblotting. LDLR band intensities were measured by densitometry, normalised with β-Actin and average intensities of three independent experiments ± SEM was represented; ns non-significant. **b** HepG2 cells were transiently transfected with plasmid encoding hPCSK9 and empty vector as mentioned in method section. HG medium was added for 24 h, expression of LDLR and PCSK9 was analyzed by Western blot. **c** HepG2 cells were transiently transfected with plasmid encoding hPCSK9 and empty vector as mentioned in method section. HG medium was added for 24 h, treated with DiI-LDL (10 µg/ml) for 2 h, fixed with 3% paraformaldehyde, mounted with UltraCruz® mounting medium (Santa Cruz Biotechnology) and images were acquired on Zeiss LSM 510 microscope.
