# Supplementary material for: Hepatocellular carcinoma-associated hypercholesterolemia: involvement of proprotein-convertase-subtilisin-kexin type-9 (PCSK9)
Source: Cancer Metab. 2018 Oct 25;6:16. doi: 10.1186/s40170-018-0187-2 (PMC6201570; doi:10.1186/s40170-018-0187-2)
Supplement: Supplementary file 7 — Figure S6. Screening of sorafenib and LDLc concentration (DOCX 76 kb) [file 40170_2018_187_MOESM7_ESM.docx]

**Additional File 7: Figure S6**

**
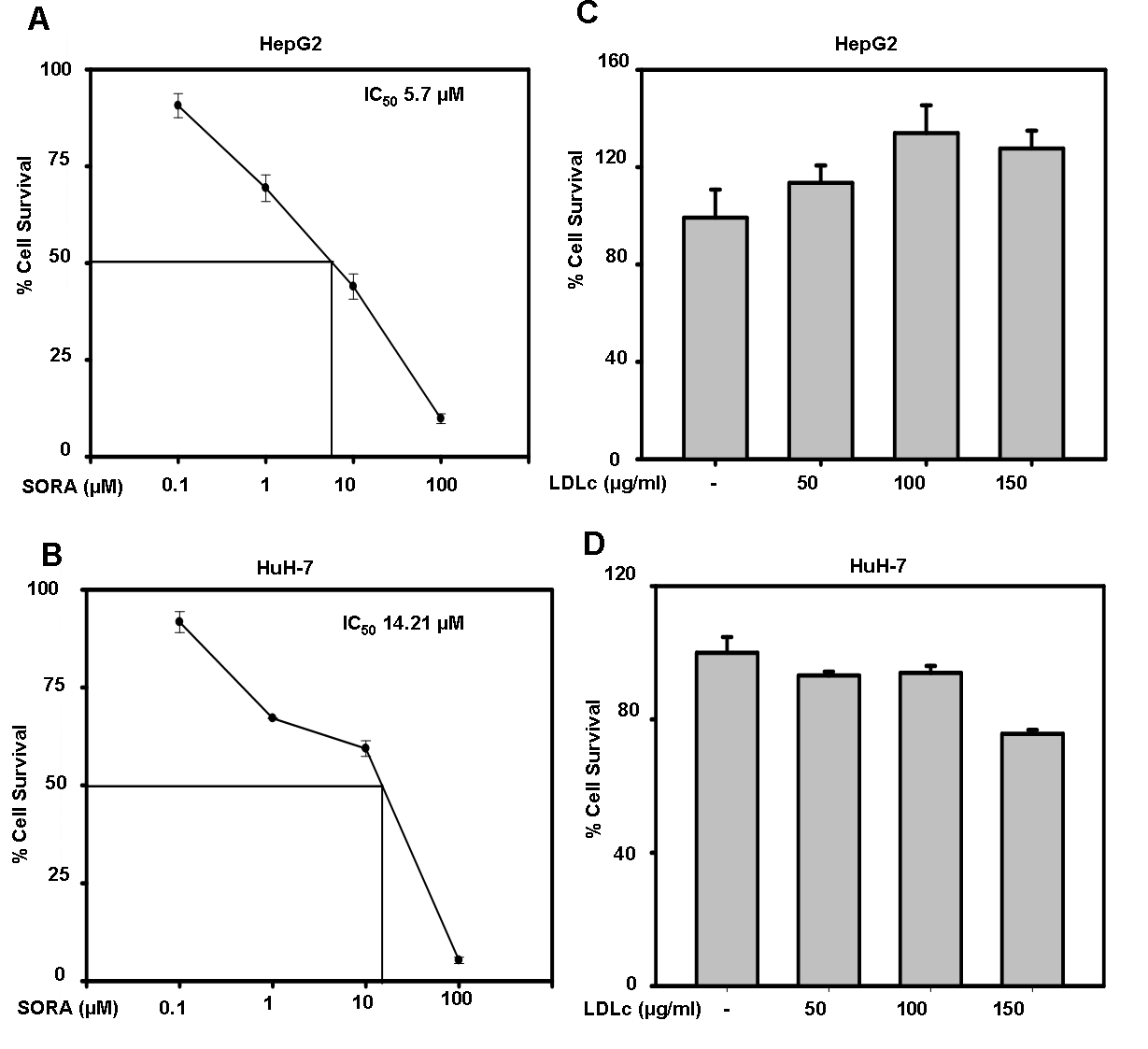
**

**Figure S6: Screening of sorafenib and LDLc concentration. a, b** HepG2 and HuH-7 cells were treated with indicated concentrations of sorafenib for 48 h, cell survival was assessed by MTT assay and IC_50_ of sorafenib was calculated by Sigma Plot 10.0. (CA, USA). **c, d** HepG2 and HuH-7 cells were treated with indicated concentrations of LDLc for 72 h and cell survival was assessed by MTT assay. The results are given as means ± standard deviation.
