# Supplementary material for: Hepatocellular carcinoma-associated hypercholesterolemia: involvement of proprotein-convertase-subtilisin-kexin type-9 (PCSK9)
Source: Cancer Metab. 2018 Oct 25;6:16. doi: 10.1186/s40170-018-0187-2 (PMC6201570; doi:10.1186/s40170-018-0187-2)
Supplement: Supplementary file 8 — Figure S7. Effect of LDLc on sorafenib mediated signaling (DOCX 112 kb) [file 40170_2018_187_MOESM8_ESM.docx]

**Additional File 8: Figure S7**

**
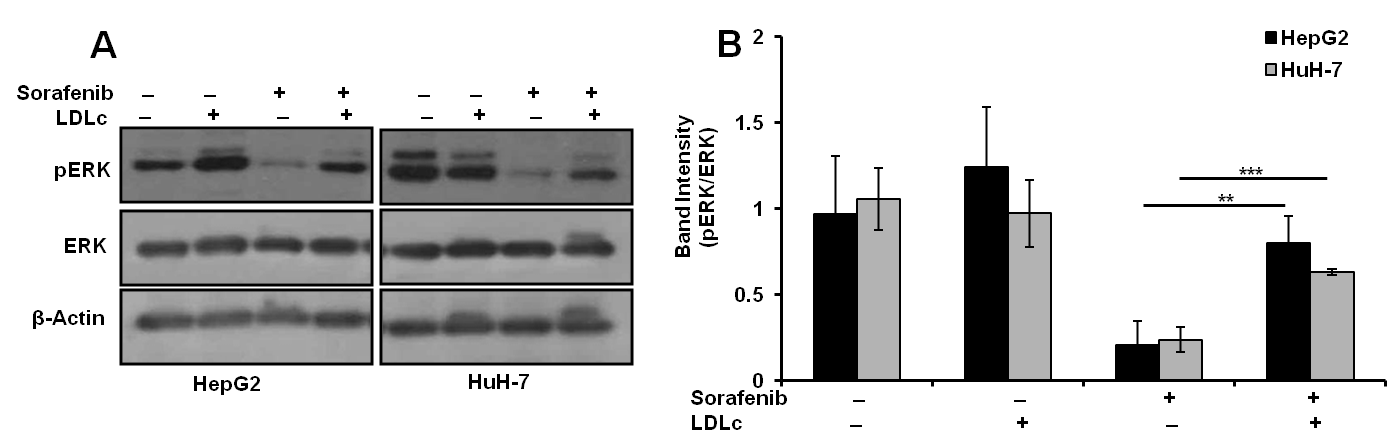
**

**Figure S7: Effect of LDLc on sorafenib mediated signalling. a** HepG2 and HuH-7 cells were seeded and allowed to adhere. Cells were cultured in DMEM containing 5% LPDS for overnight. Cells were pre-treated with LDLc (100 µg/ml) for 2 h in fresh DMEM containing 5% LPDS followed by treatment of sorafenib (5 µM) for 1 h, as indicated. Whole cell lysates were resolved on SDS-PAGE and expression of pERK, ERK and β-Actin were analyzed by Western blot. **B** Band intensities of pERK from three independent experiments were measured by densitometry and normalized with band intensities of ERK. The results are depicted in a bar graph as mean ± standard deviation; **p < 0.01 and ***p<0.001 denote significant differences in the groups.
